# Cardiomyocyte Dysfunction in Inherited Cardiomyopathies

**DOI:** 10.3390/ijms222011154

**Published:** 2021-10-15

**Authors:** Roua Hassoun, Heidi Budde, Andreas Mügge, Nazha Hamdani

**Affiliations:** 1 Institut für Forschung und Lehre (IFL), Molecular and Experimental Cardiology, Ruhr University Bochum, 44801 Bochum, Germany; Roua.Hassoun@rub.de (R.H.); heidi.budde@rub.de (H.B.); andreas.muegge@ruhr-uni-bochum.de (A.M.); 2 Department of Cardiology, St. Josef-Hospital and Bergmannsheil, Ruhr University Bochum, 44801 Bochum, Germany

**Keywords:** inherited cardiomyopathies, sarcomeric proteins, mutations, cardiomyocyte mechanics

## Abstract

Inherited cardiomyopathies form a heterogenous group of disorders that affect the structure and function of the heart. Defects in the genes encoding sarcomeric proteins are associated with various perturbations that induce contractile dysfunction and promote disease development. In this review we aimed to outline the functional consequences of the major inherited cardiomyopathies in terms of myocardial contraction and kinetics, and to highlight the structural and functional alterations in some sarcomeric variants that have been demonstrated to be involved in the pathogenesis of the inherited cardiomyopathies. A particular focus was made on mutation-induced alterations in cardiomyocyte mechanics. Since no disease-specific treatments for familial cardiomyopathies exist, several novel agents have been developed to modulate sarcomere contractility. Understanding the molecular basis of the disease opens new avenues for the development of new therapies. Furthermore, the earlier the awareness of the genetic defect, the better the clinical prognostication would be for patients and the better the prevention of development of the disease.

## 1. Introduction

**Figure 1:**
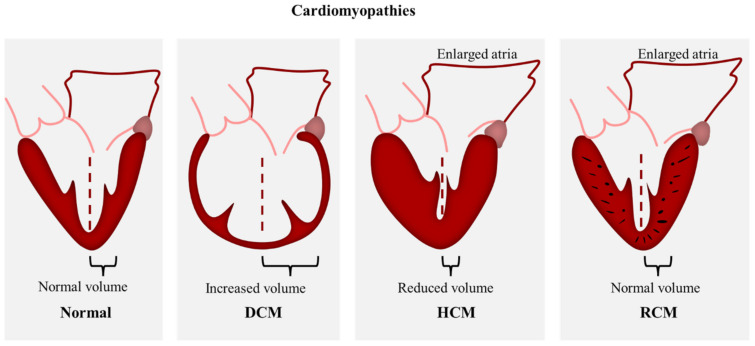
Scheme demonstrating the morphological features of HCM, DCM, and RCM.

**Figure 2:**
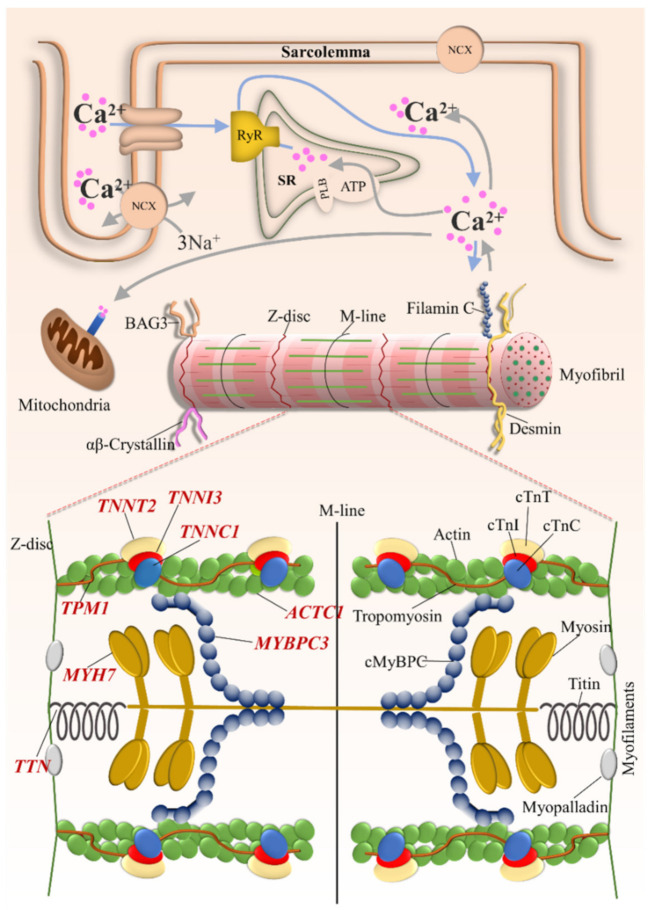
Schematic representation of the cardiomyocyte demonstrating sarcomeric genes with subcellular localization of the encoded protein. *TNNT2* = cardiac troponin T2; *TNNI3* = cardiac troponin I; *TNNC1* = cardiac troponin C; *TPM1* = α-tropomyosin; *TTN* = titin; *ACTC1* = α-cardiac actin; *MYH7* = β-myosin heavy chain; *MYBPC3* = cardiac myosin binding protein C.

Genetically inherited cardiomyopathies form a heterogeneous group of cardiac disorders characterized by structural and/or functional disturbances in the myocardium. Inherited cardiomyopathies have been described as a known cause of heart failure, arrythmia, and sudden cardiac death [1,2,3]. The phenotypic manifestation and severity of clinical outcomes can vary considerably among affected individuals. As defined by Elliot et al., 2008, cardiomyopathies are classified according to functional and morphological features into four major types: hypertrophic cardiomyopathy (HCM), dilated cardiomyopathy (DCM), restrictive cardiomyopathy (RCM), and arrhythmogenic cardiomyopathy (ACM) (Figure 1). These classes are further divided into genetic/familial and non-genetic/non-familial forms [4]. Over the past two decades, numerous mutations in sarcomeric proteins have been well established as the cause of different types of inherited cardiomyopathies (Figure 2), however, the molecular mechanisms underlying the transition from the mutation to the cardiac phenotype is not fully understood.

In this review we aimed to outline the functional consequences of the major inherited cardiomyopathies in terms of myocardial contraction and kinetics, and to highlight the structural and functional alterations in some sarcomeric variants that have been demonstrated to be involved in the pathogenesis of the inherited cardiomyopathies.

## 2. Classification

A complete classification of cardiomyopathies is divided into primary and secondary. Primary cardiomyopathies which affect the heart alone, and secondary which are the results of systematic illness affecting many parts of the body [5].

In this review, we focused mainly on the pathogenic and likely pathogenic variants that are associated with the development of inherited cardiomyopathies.

Clinvar clinical significance of the genetic variants of sarcomeric proteins follows the five terms for Mendelian diseases recommended by the American College of Medical Genetics and Genomics (ACMG) [6], in which the genetic variants are classified into five categories: “pathogenic”, “likely pathogenic”, “uncertain significance”, “likely benign”, and “benign”. This classification is based on criteria that include typical types of variant evidence such as population data, computational data, functional data, and segregation data [7]. Furthermore, the impact of the genetic variant depends on evolutionary conservation of an amino acid, the location within the protein, and the biochemical consequence of the amino acid substitution [7]. A genetic variant is considered pathogenic when the sequence variation is previously reported as disease-causing, whereas the likely pathogenic variants are those which are unreported but depending on the evolutionary conservation of the targeted amino acid, are predicted to cause the disease [6]. Variants with benign or likely benign classification are unreported to cause the disease and are expected to be non-pathogenic or probably non-pathogenic. The variant with uncertain significance refers to a variant that has an uncertain classification in the database.

### 2.1. Hypertrophic Cardiomyopathy (HCM)

HCM is a common autosomal dominant cardiac disorder with an estimated prevalence of 1:200 [1] and where more than 60% of patients show familial inheritance [8]. HCM is characterized by concentric wall thickening that mostly involve the interventricular septum and the left ventricle, causing a decrease in the chamber volume (Figure 1). The morphological features of HCM include myocyte hypertrophy, disarray, and fibrosis. Along with the reduced chamber volume, these hallmarks are known to cause diastolic dysfunction that manifest in heart failure and sudden cardiac death [2,9,10] whereas, the left ventricular systolic dysfunction was shown to be relatively rare [11]. Molecular and genetic analysis revealed the involvement of more than 1400 sarcomeric protein mutations in HCM pathogenesis, hence HCM has been considered a disease of the sarcomere [12]. These mutations were identified in genes encoding for cardiac troponin, cardiac myosin binding protein C, cardiac myosin, cardiac actin, a-tropomyosin, and titin, however genes encoding for β-cardiac myosin heavy chain, cardiac myosin binding protein C, and cardiac troponins T and I are known to be the most frequent sites of HCM mutations [13].

### 2.2. Dilated Cardiomyopathy (DCM)

DCM is characterized by systolic dysfunction and ventricular dilation in one or both ventricles [14] with an estimated prevalence of 1:250–400 and up to 1:2500 in the general population [15] (Figure 1). The clinical presentation includes arrythmia and sudden cardiac death [3]. up to approximately 40% of DCM cases are caused by genetic mutations, whereas the non-genetic cases include drugs, toxins, alcohol, and viral or bacterial myocarditis [16]. Familial DCM exhibits several patterns of inheritance including autosomal dominant, X-linked, autosomal recessive and matrilinear transmission [3,17,18,19]. To date, more than 50 DCM mutations have been identified in genes encoding for cytoskeletal, sarcomere, nuclear envelope, and intracellular adhesion proteins [15,20], in addition to other variants in ribonucleic acid-binding motif protein 20 (RBM20) and BAG3 genes [21,22]. The diversity of the affected pathways adds to the complexity of DCM pathogenesis and counts for the heterogeneity in phenotype expression. Sarcomeric DCM mutations include myosin, actin, titin, troponin, and tropomyosin [23].

### 2.3. Restrictive Cardiomyopathy (RCM)

RCM is the least common form of the cardiomyopathies. It is characterized by increased ventricular stiffness and severe diastolic dysfunction with normal wall thickness (Figure 1). The systolic function is preserved; however, it was found to be impaired at advanced stages of the disease [24]. The increase of ventricular end-diastolic pressures and atrial dilation are responsible for diastolic dysfunction and the subsequent development of progressive heart failure symptoms. Multiple inherited and acquired factors result in RCM phenotype, however, most causes of RCM are acquired. These include conditions that stiffen the myocardium such as infiltration, fibrosis, and storage diseases [25,26]. Since inherited RCM is a rare disease, the genetic and molecular understanding of its pathology is limited. Familial RCM is usually passed on in an autosomal dominant inheritance, other inheritance patterns, such as autosomal recessive and compound heterozygous, were also reported. It has been shown that the RCM phenotype can co-exist with HCM in the same families [27,28]. Furthermore, they can share some genetic mutations in sarcomeric proteins including troponin I (*TNNI3)*, β-myosin heavy chain (*MYH7)*, and myosin light chain-2 (*MYL2)* variants. Distinct sarcomeric and non-sarcomeric mutations were found to be associated with inherited RCM such as troponin T (*TNNT2*), alpha cardiac actin (*ACTC*), myosin-binding protein C (*MYBPC3*), tropomyosin 1 (*TPM1*), myosin light chain 2 (*MYL2*) and 3 (*MYL3*), αβ crystallin, desmin, filamin C, and BAG3 [29].

### 2.4. Arrhythmogenic Cardiomyopathy (ACM)

ACM is a hereditary cardiac disorder with an estimated prevalence of 1:1000 to 1:5000 in the general population [30]. The main feature of ACM is the replacement of the ventricular myocardium with fibrofatty tissue, predominantly in the right ventricle but also the left ventricle can be affected [31]. It manifests clinically in a progressive disease with ventricular dilation, systolic dysfunction, and ventricular arrhythmia that can lead to sudden cardiac death [32]. ACM is a familial disease with an autosomal dominant mode of inheritance. Most of the causative mutations were found in desmosome genes (*JUP*, *DSP*, *PKP2, DSG2*, *DSC2*). Other genetic mutations include the composita area proteins, *DES*, *PLN*, *TGFB3*, and *SCN5A*. Among the sarcomeric proteins, mutations in titin were found to cause ACM [32].

## 3. Cardiomyopathy Causative Mutations in Sarcomeric Proteins

### 3.1. The Thick Filament

#### 3.1.1. Myosin

Myosin is a large ATPase consisting of two heavy chains and four light chains. Each heavy chain consists of a rod-like tail, extruding rod (sub-fragment S2) and a globular head (sub-fragment S1) with the light chains. The head possesses a catalytic center with ATPase activity and actin binding sites. Along the tail region, myosin binds titin and myosin binding protein C (MyBP-C). MyBP-C also binds to the S2 sub-fragment. In human cardiac muscle, two isoforms are expressed. β-MyHC is expressed predominantly in the ventricles and α-MyHC in the atria. Along with MyBP-C mutations, mutations in β-MyHC gene (*MYH7*) (recently reviewed in [33]) are a frequent cause of HCM [34]. Investigations on early-onset HCM mutations (D239N and H251N) reported the increase in myosin ATPase activity, actin gliding velocity and intrinsic force [35]. Studies on different HCM mutations using highly purified recombinant human β-cardiac myosin short Subfragment 1 (sS1), showed smaller changes in myosin biomechanical parameters, suggesting that these subtle alterations alone are unlikely to give rise to the HCM phenotype [36,37,38]. Another important effector through which hypercontractility can develop, is the altered intramolecular myosin interactions responsible for keeping myosin heads in a folded-back sequestered state [39]. Mutations that occur at the head–tail (R249Q, H251N) and head–head (D382Y, R719W) interfaces have been suggested to disrupt intramolecular interactions within the sequestered state and cause more heads to become available for interaction with actin, thus leading to hypercontractility [40]. A recent study on the P710R mutation demonstrated that, even mutations that are not located in head–head or head–tail interface, might affect the ability of myosin to form the sequestered state via allosteric changes [41]. Furthermore, HCM mutations in myosin light chains have been associated with structural and functional defects in myosin [42]. In contrast to HCM mutations, DCM mutations diminish myosin activity. Biochemical characterization and mechanochemical cycle modelling of recombinant human β-cardiac myosin motor domains carrying five DCM mutations demonstrated that each mutation has a distinct effect on individual steps in the ATPase cycle depending on the location of each mutation. However, they show a common pattern on the overall cycle [43]. The DCM mutations lower occupancy of the force-holding A·M·D state (i.e., they accelerate myosin–actin detachment during cross-bridge cycle) [43,44]. The reduction in the number of myosin heads bound to actin leads to reduced work output and contractile function [45].

#### 3.1.2. Myosin Binding Protein C

MyBPC is a key regulatory protein contributing to thick filament stability and the modulation of muscle contraction. There are three different isoforms, fast skeletal, slow skeletal and cardiac, encoded by three different genes. Each isoform comprises seven Ig and three fibronectin type III domains. The cardiac isoform (cMyBPC), encoded by *MYBPC3* gene, has some specific additions. The N-terminal C0 domain, the conserved linker between C1 and C2, and a 28-amino acid loop in the C5 domain. Through its N-terminal domain, cMyBPC binds to the helical myosin S2 region, the actin filament and troponin complex [46]. The C-terminal region of cMyBPC binds the backbone region of myosin and titin. cMyBPC modulates the cardiac contractility in response to adrenergic stimulation and its subsequent phosphorylation by protein kinase A (PKA) [47,48]. In contrast to HCM, *MYBPC3* mutations fond in DCM and RCM patients are rare [29,49,50] (reviewed in [51,52]). The vast majority of HCM-linked mutations in *MYBPC3* are truncating mutations [34] including frameshift, nonsense, or conserved RNA splice site mutations. cMyBPC-truncated variants were strongly associated to allelic imbalance and haploinsufficiency leading to HCM phenotype [52]. Although HCM-linked missense (non-truncating) mutations in *MYBPC3* are associated with more benign outcomes, functional and structural investigations of a subset of missense mutations reported disturbed cMyBPC architecture and protein–protein interactions [53] suggesting a protein poison peptide as a potential pathomechanism. However, recent have studies reported RNA splicing defects, resulting from premature stop codon, and protein destabilization cause protein haploinsufficiency in *MYBPC3* missense mutations [54,55].

cMyBPC haploinsufficiency is strongly associated with enhanced Ca^2+^ sensitivity. The latter was attributed to hypophosphorylation of troponin I secondary to mutation-induced dysfunction [56]. Another mechanism through which haploinsufficiency contributes to contractile disfunction is the perturbed dynamic myosin conformations. Recent studies using mice models demonstrated that cMyBPC deficiency enhances the myosin state that enables ATP hydrolysis and thin filament interactions (DRX) and reduces the super relaxed conformation associated with low energy consumption (SRX) [54].

### 3.2. Titin

The giant protein titin, encoded by titin gene *TTN*, is a fundamental sarcomeric protein serving as a biological spring. Titin elasticity regulates the mechanical compliance of the heart through the modulation of passive tension, systolic and diastolic function, and length-dependent activation [57]. The titin molecule expands from the Z-disk (N-terminus) to the M-band (C-terminus) region of the sarcomere and comprises four domains. The N-terminal domain anchors titin to the Z-disc. The I-band domain includes the highly elastic immunoglobulin-like (Ig) domains, PEVK region (rich in proline, glutamate, valine, and lysine), N2A, and/or N2B element providing titin its spring-like function. The rigid, inextensible A-band domain containing FNIII and Ig-domains, binds to the thick filament proteins. The C-terminal domain embeds titin to the M-band [58]. Cardiac muscle works under nonequilibrium conditions—it is stretched (and released) one or many times per second. Diastolic force development is not purely elastic but has long been known to include stretch velocity-sensitive components of viscous and/or viscoelastic nature, but both passive force and dynamic stiffness principally reflect the intrinsic viscoelastic properties of titin [58]. The elastic-force component of cardiac myofibrils can be described in terms of the entropic-spring properties of titin segments, but these do not explain passive-force decay of cardiac myofibrils following quick stretch (stress relaxation), which is mainly determined by a slow viscoelastic component due to unfolding of the Ig domains and by a fast viscous drag due mainly to the interaction between titin and actin.

Missense point mutations in *TTN* are associated with different types of cardiomyopathies. RCM-causing mutation was found to be located at the highly conserved I/A junction, however the molecular mechanism of the disease progression remains elusive [59]. A few HCM associated mutations were identified, mostly at the Z-disc and I-band region, [60] and were shown to increase the binding affinity of α-actinin to titin, thereby inducing maladaptive responses to the impaired force transmission during contraction. ACM-causing mutations in titin are distributed all over the molecule [61]. Taylor et. al., 2011 suggested that the initiation of the disease is promoted via titin proteolysis, as ACM titin mutations lower the structural stability of Ig domains and lead to titin degradation [61]. Titin missense variants were also found in some cases of DCM and were expected to contribute to the disease [62]. Most pathogenic *TTN* mutations are heterozygous truncating variants resulting from frameshift, nonsense, and essential splice site mutations predominantly within A-band titin (reviewed in [63]). Titin truncating variants count for 25% of familial DCM cases [63,64,65] and are shown to be present in approximately 2% of the general population without distinct cardiomyopathy [66]. The effects of titin-truncated variants (TTNtv) are position dependent. In healthy carriers, the majority of TTNvs mutations were identified in the I-band exons. Mutations in the I-band exons are exposed to alternative splicing, whereas the A-band includes a vast array of constitutively expressed exons, hence, mutations at the I-band region are better tolerated [65].

TTNvs-based DCM is associated with a late onset, however, when combined with additional stressors and risk factors, they show earlier presentation. The mechanism by which TTNvs contributes to DCM is not fully understood, however, several mechanisms have been suggested to play an important role in DCM pathology. Haploinsufficiency was reported in animal model studies showing the increase of nonsense-mediated mRNA decay (NMD), titin insufficiency and a subsequent sarcomere disassembly [67,68]. Other studies suggested that the disease can be initiated via poison peptide mechanism rather than haploinsufficiency [65]. In patient-derived iPSC cardiomyocytes, *TTN* was found to be biallelically expressed [69]. Furthermore, and due to the loss of a β-cardiac myosin binding site on the mutated titin protein, abnormal sarcomerogenesis and impaired force generation were also detected in these cells [70]. Impaired signaling pathways and cardiac metabolism have been shown to be correlated with TTNvs-based DCM. Adams et al., 2019, reported the increase in ROS generation and mitochondrial dysfunction in rat hearts with TTNtv [71], in addition to the increase in the mammalian target of rapamycin (mTOR) phosphorylation and impaired autophagy.

### 3.3. The Thin Filament

#### 3.3.1. Actin

Actins are highly conserved proteins among the species, they are classified into three groups of isoforms (α-actin, β-actin, and γ-actin) encoded by six different genes. α-cardiac actin, encoded by *ACTC1*, is expressed predominantly in cardiac muscle. Under physiological conditions, the globular monomeric G-actin polymerizes to form a double helical filamentous structure (F-actin). The interaction of filamentous actin with β-myosin and other components of the contractile apparatus is essential for muscle contraction. Hence, cardiac actin mutations that alter this interaction are known to impair myocyte structure, cross-bridge cycling, and force generation. More than 50% of *ACTC1* mutations are HCM-causing mutations and about 20% are DCM-causing mutations, however, they occur with an incidence of 4–6% in patients with familial cardiomyopathy. To date, twelve missense dominant mutations in *ACTC1* have been reported to cause familial HCM (Table 1), and two to cause DCM [72]. Amino acid replacements that alter actin binding affinity towards myosin, such as E99K, were shown to affect acto–myosin interaction and force generation, leading to compensatory hypertrophy [73,74]. Other mutations were shown to affect acto–myosin interaction indirectly, such as the A295S variant that has been associated with HCM. The position of amino acid substitution affects the tropomyosin-blocked-state binding side on actin resulting in altered thin-filament activation, increased Ca^2+^ sensitivity, and hypercontractility [75]. E361G, a DCM-causing mutation, was demonstrated to uncouple β1-adrenergic signaling and affect Ca^2+^ sensitivity and troponin I phosphorylation [72]. Moreover, the DCM-causing R312H mutation was reported to affect actin–tropomyosin interaction and decrease the structural stability of thin filaments [76]. In a recent study by us, we found that both DCM-causing mutations (E361G and R312H) exhibited reduced rates of MICAL-induced depolymerization, indicating an increased persistence of these variants into sarcomeric structures which might potentially affect cardiomyocyte differentiation and function [77]. We have also shown that these variants incorporate differently into sarcomeric thin filaments which might lead to mechanical instability and sarcomere disturbances [77].

#### 3.3.2. Tropomyosin

Tropomyosin (Tm) is an actin-binding regulatory protein. There are three groups of isoforms (α-Tm, β-Tm, and γ-Tm) encoded by different genes. α-Tm, encoded by *TPM1*, is the major isoform expressed in the heart. It consists of two parallel α-helical coiled coils that span seven actin monomers forming a continuous homo or heterodimer [86]. Through its interaction with the heterotrimeric troponin complex, Tm plays an essential role in regulating the Ca^2+^-induced actin–myosin interaction [87,88,89]. Point-mutations in *TPM1* have been discovered in patients with HCM and DCM (Table 2). Functional and structural analysis revealed altered interaction within Tm coiled coil and between Tm and actin [90]. The disturbed actin–Tm interaction affects the binding of the myosin head and results in altered cross-bridge kinetics [91]. Altered Tm–troponin interaction affects the three-state functioning of tropomyosin during thin filament activation [86,92]. Furthermore, mutation-induced-changes in tropomyosin flexibility were shown to modulate the Ca^2+^-dependent-activation cooperativity of the thin filament [93]. Redwood and Robinson 2013, suggested that the changes in Ca^2+^-regulation due to mutations, stimulate hypertrophic signaling pathways [94]. Memo et. al., 2013 reported that DCM mutations uncouple the effect of PKA-dependent troponin phosphorylation on Ca^2+^ sensitivity [95]. Hence, impaired myocardial contractility appears to be the primary effect of Tm mutations [94].

#### 3.3.3. Troponin Complex

Cardiomyocyte contraction occurs as a result of cell membrane depolarization and subsequent elevation in cytosolic calcium level. The key role in the regulation of muscle contraction belongs to the troponin complex (Tn), discovered by Ebashi and Kodama, 1965 [112]. This protein consists of three subunits, troponin T (the Tpm-binding subunit, TnT), troponin I (the inhibitory subunit, TnI) which inhibits the ATPase activity of actomyosin complex in the absence of Ca^2+^, and troponin C (the Ca^2+^-binding subunit, TnC) [86,113,114].

##### Troponin C

The cardiac/slow-twitch TnC (cTnC) isoform is encoded by *TNNC1* gene, while *TNNC2* encodes for fast skeletal isoform (fsTnC). cTnC is a member of the EF hand family of Ca^2+^-binding proteins. It has a dumbbell-shaped molecule that consists of two globular N- and C-terminal domains connected by a central helical linker. The two C-terminal high-affinity Ca^2+^-binding domains (EF3 and EF4) can bind either Ca^2+^ or Mg^2+^ which enhances the interaction of cTnC with cTnI and stabilizes the cTn complex. The N-terminal regulatory domain EF1 is a nonfunctional domain, while the domain EF2 has a low affinity but high selectivity for Ca^2+^, therefore, unlike the fast skeletal isoform of TnC, which has four active Ca^2+^ binding sites, cTnC can only bind three Ca^2+^ ions [115]. Multiple mutations in *TNNC1* have been associated with HCM, DCM and RCM [116] (recently reviewed in [117]), most of which were reported to modulate cTnC function by altering the cTnC binding affinity for calcium and/or its binding partners. Mutations in the N-terminal lobe are considered to affect the conformational activation (on and off states of cNTnC), Ca^2+^ binding properties and the binding-to-cTnI switch peptide [114,118]. L29Q is a well-studied HCM-causing mutation in cTnC. However functional studies using different experimental models reported contradictory results demonstrating Ca^2+^ sensitivity to be increased [119], decreased [120] or unchanged [121]. Other HCM mutations in cNTnC, such as A8V, A31S and C84Y were strongly associated with increased Ca^2+^ sensitivity. These variants were shown to either increase the stability of cNTnC open conformation, thereby enhancing Ca^2+^-binding affinity, or to directly increase the binding with cTnI [122,123,124,125]. In a recent study by us, we provided evidence on structural and functional alterations in thin filaments containing the novel cTnC G34S variant [126]. We found the TnC G34S mutation to increase Ca^2+^-binding affinity, decrease the binding affinity towards cTnT, and to cause structural impairments in the reconstituted thin filaments. Mutations in the C-terminal lobe affect the interactions of cTnC with cTnI, cTnT, and Tpm [127]. The HCM-associated variant E145D was found to abolish Ca^2+^ binding to site IV and to increase Ca^2+^-binding to site II allosterically [128]. Furthermore, it has been demonstrated that the E145D variant alters interactions with the N-terminal domain of cTnI (cNTnI) and the C-terminal domain of cTnT (cCTnT) [129]. HCM-associated mutations in cTnC have also been linked to the development of RCM phenotypes, such as A8V, A31S, and E145D variants [130,131].

The DCM-associated mutation G159D at the cCTnC was shown to impair the interaction with cTnT, decrease myofilament Ca^2+^ sensitivity, and blunt the effect of cTnI phosphorylation at Ser 22/23 [132,133]. In this case, the structural derangements caused by this mutation seems to exceed the TnI dephosphorylation which cannot compensate for the decrease in Ca^2+^ sensitivity [134]. The double compound heterozygous DCM variant (E59D/D75Y) has also been shown to decrease myofilament Ca^2+^ sensitivity and reduce strong actin–myosin binding [135,136]. A recent study by Landim-Vieira et al., 2019 on the missense compound heterozygous DCM-causing variants (D132N and D145E) demonstrated no significant changes in contractile parameters, indicating to the potential involvement of other pathological mechanisms beyond myofilament dysfunction [137].

##### Troponin I

Troponin I is the inhibitory subunit of troponin complex. Different genes encode the fast skeletal, slow skeletal and cardiac isoforms. cTnI, encoded by *TNNI3*, consists of several functional domains, a cardiac specific N-terminal extension region (residues 1–32), an IT-arm region, the inhibitory region (residues 130–150), the switch region (residues 151–167), and the C-terminal mobile domain. Protein kinase A (PKA)-mediated phosphorylation of cTnI at Ser-23/Ser-24 reduces myofilament Ca^2+^ sensitivity, increases the rate of Ca^2+^ dissociation from cTnC and cross-bridge cycling [138]. Mutations found in *TNNI3* have been linked to DCM, RCM, and HCM (reviewed in [139]), most of which are located at the inhibitory region and C-terminal region of the protein. Families harboring D190G mutation exhibited mixed appearance of both RCM and HCM phenotypes, suggesting that some *TNNI3* mutations might cause RCM and HCM in a similar mechanism [140]. Mutations within the inhibitory region of cTnI increase the binding affinity of cardiac troponin C in the absence of Ca^2+^, thereby increasing the basal level of ATPase activity and Ca^2+^ sensitivity which contributes to the depressed relaxation in HCM and RCM [141]. HCM mutations that were found at the C-terminal domain of cTnI exhibited decreased actin–myosin interaction, consequently reducing the inhibition of basal actomyosin ATPase activity and increasing the Ca^2+^ sensitivity of muscle contraction [142,143]. Furthermore, cCTnI mutations, such as R170W, showed disturbed incorporation into the thin filaments, which might potentially lead to excess protein accumulation and aggregation in the cytosol [29]. It has been also reported that HCM mutations, such as K206Q and R145G, uncouple the effect of TnI phosphorylation by PKA [144]. Furthermore, we showed the D127Y variant to increase the Ca^2+^ sensitivity of reconstituted thin filaments, induce structural perturbations, and cause RCM phenotype [126]. In contrast to HCM and RCM mutations, DCM mutations in *TNNI3* are less frequent. A2V was the first DCM-causing mutation discovered in *TNNI3*, causing an impaired TnI–TnT interaction. Functional studies have demonstrated that K36Q and N185K mutations decrease the maximal force and Ca^2+^ sensitivity of acto-myosin-S1 ATPase leading to the development of DCM phenotype [145].

##### Troponin T

Cardiac, fast, and slow twitch isoforms of TnT are encoded by three different genes. TnT has two functional domains. The N-terminal domain T1 anchors Tn complex to the thin filament through its binding to Tm. The C-terminal domain T2 interacts with other Tn subunits (TnI and TnC) and Tm/actin. *TNNT2* encodes the cardiac TnT and undergoes alternative splicing to produce four distinct cardiac isoforms in the human heart (cTnT1-cTnT4) [146]. PKC-dependent phosphorylation of cTnT and the alternative splicing of *TNNT2* have been suggested to contribute to Ca^2+^-dependent regulation of muscle contraction [147]. Most HCM- and DCM-causing mutations in *TNNT2* (reviewed in [139]) were found in the conserved middle or C-terminal region which contain TnI, TnC and tropomyosin binding sites [148]. Patients carrying HCM mutations in *TNNT2* exhibited variable clinical outcomes and prognosis. Several mutations such as I179N, R92Q, R92W, R94L induced malignant phenotypes, however they were highly associated with sudden cardiac death and a short life span. Functional studies revealed the increase in Ca^2+^ sensitivity of myofilament force production. They suggested the interaction between TnT and Tm to be impaired leading to decreased inhibitory function of TnI on actin [149]. RCM-causing mutations in *TNNT2* are rare. They demonstrated increased Ca^2+^ sensitivity and diminished ATPase inhibition and relaxation [29,150]. DCM-causing mutations (R131W, R141W, R205, ΔK210, R205L, and D270N) were shown to decrease Ca^2+^ sensitivity of force generation in skinned fibers [139]. R141W variant was reported to enhance TnT–Tm binding affinity leading to strong inhibitory effect of cTnI on the thin filament, and hence Ca^2+^ desensitization.

## 4. Altered Cardiomyocyte Forces and Kinetics in Inherited Cardiomyopathies

### 4.1. Ca^2+^ Sensitivity

Accumulating evidence has supported the association between altered Ca^2+^ sensitivity and inherited cardiomyopathies. Functional studies on sarcomeric mutations demonstrate that HCM and RCM mutations increase myofilament Ca^2+^ sensitivity and active force generation, while DCM mutations reduce it [151], hence opposing mechanical mechanisms contribute to the development of HCM, RCM and DCM [152]. The increased Ca^2+^ sensitivity of force development has been suggested to be the primary defect induced by HCM and RCM mutations. In general, higher Ca^2+^ sensitivity was detected in RCM compared with HCM mutations [153]. Several studies report HCM-associated mutations in sarcomeric proteins, especially in myosin [154,155], Tm [92] and cMyBPC [156,157], to cause perturbed myosin dynamics and disruption in the inhibitory regulation of actin leading to increased Ca^2+^ sensitivity. Such disturbances are attributed to mutation-induced-alterations in protein–protein interactions or protein haploinsufficiency, as discussed earlier in this review.

**Figure 3:**
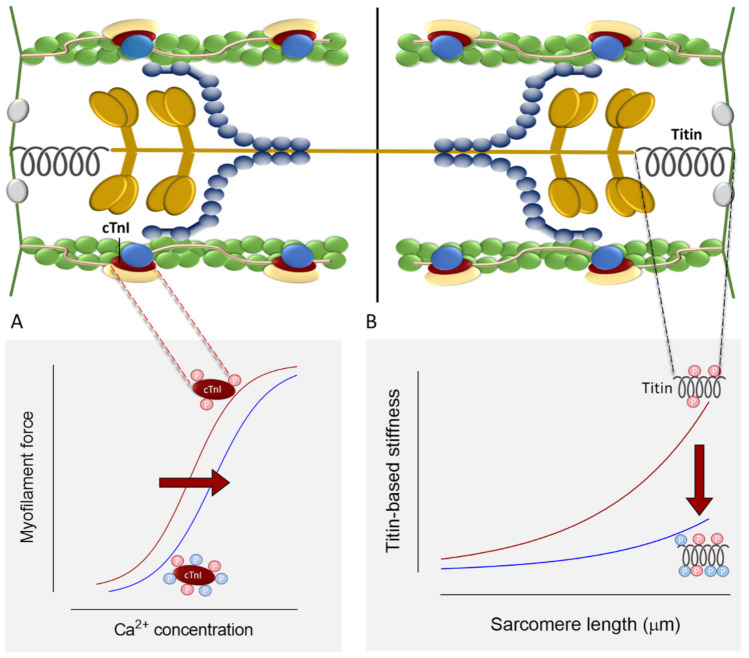
(**A**). Effects of cTnI phosphorylation on myocyte Ca^2+^ sensitivity. (**B**). Effects of titin phosphorylation on myocyte passive stiffness.

Perturbed actin–myosin attachments can strongly affect Ca^2+^ sensitivity. In vitro motility assays using thin filaments reconstituted with actin variants, such as R312H, showed altered myosin-driven motility and Ca^2+^ sensitivity [72]. Such alterations were attributed to the mutation-induced alterations in tropomyosin–actin interaction which inhibits strong actin–myosin binding in the absence of Ca^2+^ [72]. Furthermore, tropomyosin mutations that affect its binding with the myosin head, such as D137L and G126R, were shown to increase the maximal sliding velocity of regulated actin filaments in the in vitro motility assay at high Са^2+^ concentrations and to increase the Са^2+^ sensitivity of the actin–myosin interaction [158]. It has been suggested that theses mutations facilitate the displacement of tropomyosin from actin by a strong bond with myosin heads, allowing more myosin heads to bind actin, and hence increasing force production [158]. Another modulator of Ca^2+^ sensitivity is PKA-mediated phosphorylation of cTnI and cMyBPC (Figure 3). As shown in previous studies using HCM and end-stage heart failure myocardial samples, the increased Ca^2+^ sensitivity was correlated with reduced phosphorylation levels of cTnI and cMyBPC and could be reversed upon PKA treatment [159,160,161]. In addition to PKA, PKC- and PKD-dependent phosphorylation of cTnI modulates Ca^2+^ sensitivity [162]. Structural and functional investigations suggested that, depending on the position of RCM and HCM mutations in cTnI, they either impair the accessibility of the kinases [163,164], or blunt the functional response upon phosphorylation [165]. Furthermore, the structural derangements caused by DCM mutations were found to uncouple troponin I phosphorylation from Ca^2+^ sensitivity changes [72,95]. Hence the altered response to β-adrenergic signaling and downstream phosphorylation of sarcomeric proteins play a major role in determining the myofilament Ca^2+^ sensitivity. Furthermore, the length-dependent increase in Ca^2+^ sensitivity is found to be reduced in human cardiac samples from patients carrying HCM and DCM mutations [166]. Length-dependent activation (LDA) is the main mechanism responsible for the Frank–Starling effect [167]. The perturbed LDA contributes to the myofilament dysfunction associated with DCM and HCM mutations.

On the cellular level, enhanced Ca^2+^ sensitivity was shown to increase Ca^2+^-binding affinity to the thin filament [168,169], causing delayed relaxation, hypercontractility, increased ATP utilization and arrhythmia [5]. On the other hand, Ca^2+^ desensitization in DCM accounts for systolic dysfunction and the initiation of ventricular dilation. The latter develops as a compensatory response to the decrease in stroke volume [5].

### 4.2. Cross-Bridge Kinetics

The basal acto-myosin-S1 ATPase activity was found to be enhanced in skinned fibers containing RCM and HCM mutations in cTnI [153,170], indicating the depressed relaxation properties of the myofilament which leads to the diastolic dysfunction. As previously discussed in this review, mutations in thick filament proteins that affect the sequestered state conformation of myosin alters the ATPase cycle and cross-bridge kinetics leading to hypocontractility (DCM) or hypercontractility (HCM and RCM). Both of which are associated with impaired systolic function. HCM mutations were found to be associated with reduced maximum Ca^2+^ activated force. Hoskins et al., 2010 suggested the structural alterations in myosin cause fast cross-bridge detachment leading to a loss in maximal force generation and a subsequent systolic dysfunction in HCM myocytes [161]. However, the study attributed the reduction in maximal force to changes secondary to the mutations, as no loss of myofibrils was detected [161]. Studies of animal models and human myocardial samples of HCM revealed the increase in ATP utilization for force generation (increased tension cost) [171,172]. ATP depletion impairs Ca^2+^ uptake via the high energy-dependent pump of the sarcoplasmic reticulum (SERCA2), leading to elevation in cytosolic free Ca^2+^ and Ca^2+^-dependent signaling. DCM-linked mutations also exhibit impaired Ca^2+^ cycling. As the Ca^2+^ sensitivity is decreased in DCM, higher levels of free cytosolic Ca^2+^ are required for tension generation, and Ca^2+^ dissociates rapidly from sarcomeres during relaxation [173]. The impaired Ca^2+^ cycling counts for the activation of signaling pathways that promote cardiac remodeling and growth [174].

### 4.3. The Myocyte Passive Stiffness

Interstitial fibrosis and myocyte disarray are responsible for ventricular stiffness observed in HCM and RCM [175]. However, myocardial stiffness can arise from the intrinsic stiffness of the myocyte itself. Titin is the major determinant of myocyte passive stiffness. Titin stiffness was shown to be positively correlated with active force in HCM and DCM [176]. HCM mutations lead to an increase in titin stiffness [177], whereas a decrease in titin stiffness was observed in DCM mutations [178]. These alterations are attributed to titin isoform switching. The increased expression of the compliant titin isoform (N2BA) associates with reduced myocardial stiffness and sarcomere rigidity found in eccentric remodeled hearts with systolic dysfunction, such as DCM [179,180,181], whereas reduced N2BA:N2B expression ratios were observed in HCM [182,183]. Such alterations could follow the primary defects caused by sarcomeric mutations. It has been previously demonstrated that during the progression of heart failure, the maladaptive response leads to the upregulation of N2BA at the expense of the N2B isoform, causing reduced passive stiffness, increased end diastolic volume, and hence ventricular dilation [178]. Along with isoform transitions, titin compliance is modulated by post-translational modifications including phosphorylation (Figure 3) [184]. In general, phosphorylation of the negatively charged N2Bus leads to a decrease, and phosphorylation of the positively charged PEVK leads to an increase of passive stiffness [58,185]. Previous studies have reported the altered PKA-, PKG-, PKC-, and CAMKII-dependent phosphorylation of titin, and hence the altered myocardial stiffness in DCM and HCM [177,186].

## 5. Therapies

As no disease-specific treatments for familial cardiomyopathies exist, the management of the disease includes combination therapies to prevent heart failure progression and sudden cardiac death. Several novel agents have been shown to modulate sarcomere contractility such as the cardiac myosin inhibitor mavacamten (MYK-461). In HCM mice model carrying the *MYH7* mutation, mavacamten was shown to reduce the ATPase activity of myosin, attenuate hypercontractility and profibrotic gene expression, and enhance relaxation [187]. Ca^2+^ sensitivity has also been targeted via agents that modulate actin–myosin interaction. Blebbistatin was shown to stabilize the thick filament off state and reduce actin-myosin cross-bridge formation and arrhythmia susceptibility in HCM [188,189]. Cardiac myosin activators such as omecamtiv mecarbil exhibited beneficial effects in DCM [190]. Omecamtiv mecarbil accelerates the transition of myosin from the weakly into the strongly actin-bound without altering the Ca^2+^ transient [191]. Another group of agents that modulate Ca^2+^ sensitivity is troponin-targeting agents. Levosimendan, a Ca^2+^ sensitizer, binds to the N-terminal domain of cTnC and stabilizes its open conformation [192]. Unlike most inotropes, levosimendan was demonstrated to enhance cardiac contractility without increasing oxygen demand [193] suggesting a beneficial therapeutic effect in DCM. The green tea catechin (−)-epigallocatechin gallate (EGCg) has been shown to bind to the C-terminal domain of cTnC and interfere with the cTnC–cTnI interaction thereby decreasing Ca^2+^ sensitivity in myofilaments [194]. Furthermore, EGCg improved the diastolic function in an HCM mouse model [195]. In a recent study by us, we found levosimendan and EGCg to interact directly with actin and to improve the structural integrity of thin filaments reconstituted with troponin variants [126].

Clinical trials on several sarcomere contractility modulators showed moderate improvement in cardiac function (recently reviewed in [196]). Moreover, the adverse events caused by these drugs necessitates the development of analogous that are better tolerated.

Another evolving approach for the treatment of familial cardiomyopathy is gene therapy. HCM mutations in *MYBPC3* that cause protein haploinsufficiency were managed by a viral transfer of wild-type *MYBPC3* cDNA into human iPS derived cardiomyocyte [197]. The study reports the increased levels of wild type cMyBPC and improvement in cardiac function [197]. RNA interference has been used to silence the mutant RNA in a mouse model harboring *MHY6* mutation and resulted in the prevention of HCM phenotype development [198]. CRISPR/Cas9 system in human embryos has been used to correct heterozygous mutation in *MYBPC3* [199]. The study reports high efficiency and low adverse effects on embryonic development [199].

## 6. Conclusions

Inherited cardiomyopathies are known to cause heart failure and sudden cardiac death. Although the genotype–phenotype correlation is promoted via different pathological mechanisms, there is strong evidence that mutations in the sarcomeric proteins account for several defects in the contractile function. The impaired Ca^2+^ handling and the subsequent alterations in cardiomyocyte mechanics are common initiators of the disease. Understanding the molecular basis of the disease opens new avenues for the development of new therapies. The early awareness of the genetic defect, management of the risk factors, and prophylactic strategies play an important role in the clinical prognostication and the prevention of sudden cardiac death.

## Figures and Tables

**Table 1:** Actin mutations associated with CMs.

Variant	Phenotype	References
R312H	DCM	[78]
E361G	DCM	[78]
E99K	HCM	[79]
P164A	HCM	[79]
A331P	HCM	[79]
Y166C	HCM	[80]
M305L	HCM	[80]
A295S	HCM	[81]
R314C	HCM	[82]
S271F	HCM	[83]
A232V	HCM	[84]
R97C	HCM	[85]
H90Y	HCM	[85]
F92del	HCM	[82]

**Table 2:** Tropomyosin mutations associated with CMs.

Variant	Phenotype	References
E40K	DCM	[96]
E54K	DCM	[96]
D84N	DCM	[97]
D230N	DCM	[98]
M8R	DCM	[99]
K15N	DCM	[100]
E23Q	DCM IDC	[100]
I92T	DCM	[100]
T201M	DCM	[101]
A239T	DCM	[100]
A227V	DCM idiopathic	[100]
E192K	HCM sporadic	[102]
D58H	HCM	[103]
A107T	HCM	[104]
A22T	HCM	[104]
R21H	HCM	[105]
E62Q	HCM/RCM	[106,107]
A63V	HCM	[108]
K70T	HCM	[109]
V95A	HCM	[110]
I172T	HCM	[84]
M281T	HCM/RCM	[107,84]
L185R	HCM	[84]
D175N	HCM	[12]
E180G	HCM	[12]
E180V	HCM in early stageDCM above 40 years	[111]
S215L	HCM	[85]
I284V	HCM	[83]

## Data Availability

Not applicable.
